# An assessment of Makerere University College of Health Sciences: optimizing health research capacity to meet Uganda’s priorities

**DOI:** 10.1186/1472-698X-11-S1-S12

**Published:** 2011-03-09

**Authors:** Ziadah Nankinga, Paul Kutyabami, Dan Kibuule, Joan Kalyango, Sara Groves, Robert C Bollinger, Celestino Obua

**Affiliations:** 1 School of Health Sciences, College of Health Sciences, Makerere University, Kampala, Uganda; 2 School of Biomedical Sciences, College of Health Sciences, Makerere University, Kampala, Uganda; 3 Johns Hopkins School of Medicine, Baltimore, Maryland, 21205, USA; 4 Johns Hopkins School of Nursing, Baltimore, Maryland, 21205, USA

## Abstract

**Background:**

Health research is critical to the institutional mission of the Makerere College of Health Sciences (MakCHS). Optimizing the alignment of health research capacity at MakCHS with the health needs and priorities of Uganda, as outlined in the country’s Health Sector Strategic Plan (HSSP), is a deliberate priority, a responsibility, and a significant opportunity for research. To guide this strategic direction, an assessment of MakCHS’s research grants and publication portfolio was conducted.

**Methods:**

A survey of all new and ongoing grants, as well as all publications, between January 2005 and December 2009 was conducted. Research, training, and education grants awarded to MakCHS’ constituent faculties and departments, were looked for through financial records at the college or by contact with funding organizations. Published manuscripts registered with PubMed, that included MakCHS faculty authors, were also analyzed.

**Results:**

A total of 58 active grants were identified, of which 18 had been initiated prior to 2005 and there were an average of about eight new grants per year. Most grants funded basic and applied research, with major focus areas being HIV/AIDS (44%), malaria (19%), maternal and child health (14%), tuberculosis (11%), mental health (3%), and others (8%). MakCHS faculty were identified as Principal Investigators (PIs) in only 22 (38%) active grants. Grant funding details were only available for one third of the active grants at MakCHS. A total of 837 publications were identified, with an average of 167 publications per year, most of which (66%) addressed the country’s priority health areas, and 58% had MakCHS faculty or students as first authors.

**Conclusions:**

The research grants and publications at MakCHS are generally well-aligned with the Ugandan Health Ministry priorities. Greater efforts to establish centralized and efficient grants management procedures are needed. In addition, greater efforts are needed to expand capacity for MakCHS faculty leadership of grants, as well as to continue to expand the contribution of MakCHS faculty to lead research publications.

## Background

The Makerere University College of Health Sciences (MakCHS) has a mandate to provide training and quality services, as well as to conduct research for improving the health of the people of Uganda and beyond. Health research is a critical part of the historic and institutional mission of the MakCHS. Therefore, strengthening research capacity, as well as optimizing the alignment of health research at MakCHS with the health needs and priorities of Uganda, as outlined in the country’s Health Sector Strategic Plans (HSSP) is a deliberate priority, a responsibility and a significant opportunity [1,2].

In order to guide MakCHS in this strategic direction, an assessment was conducted to determine the range and impact of basic and applied research currently conducted by MakCHS. The assessment indentified the number of MakCHS grants and publications, as well as the extent of involvement of MakCHS faculty in their leadership and management. Identifying opportunities to optimize health research impact and relevance is valuable to MakCHS, as well as other institutions in Africa and around the world.

## Methods

### Assessment of research grants at MakCHS

An assessment of all new and ongoing grants for research, training, and education awarded to MakCHS’ constituent faculties and departments between January 2005 and December 2009 was conducted. The authors developed a data collection instrument that included: the principal investigator, sponsors, grant duration, funds awarded, the school/department receiving the grant, and the name of the grant. Based on the description and information available on the grants, they also categorized them into one of four areas: basic and applied research, training, capacity building, and educational administrative support. Grants were also examined for alignment with HSSP priority areas. A primary topic from a list of HSSP priority areas was assigned to each grant, based on an assessment of the major scope of the grant. These topic areas included malaria, HIV/AIDS, tuberculosis, maternal and child health, environmental health, and mental health [2].

MakCHS initial list of past funding sources were obtained from the accounts office that handles its funding. The list was subsequently expanded by the Johns Hopkins University Welch Medical Library librarians who conducted searches of additional potential sources of funding for MakCHS in the last five years. Lastly, funding agencies were directly contacted regarding grant information. These included: African Malaria Network Trust, Bill and Melinda Gates Foundation, Canadian International Development Agency, Carnegie Corporation, Department for International Development, Doris Duke Charitable Foundation, Ford Foundation, Norwegian Agency for Development Cooperation, Pfizer, the Rockefeller Foundation, Swedish International Development Agency, U.S. Agency for International Development, U.S. Department of Health and Human Services Maternal and Child Health Bureau’s Discretionary Grant Information System, and **U.S.** National Institutes of Health. Each of these funding sources were contacted via e-mail to confirm funding amounts for projects or research at M**ak**CHS in the study period. Local and international collaborating institutions were also contacted for grants that might have been provided during the same time period. Additional in depth internet searches were done to clarify information about the grants identified to determine the type, amount and the status of such grants, and the principal investigator.

In this study, the grants analyzed included basic and applied research grants, as well as educational and capacity building grants to the College. Individual fellowships where the funding was not accessed through MakCHS institutions were excluded. A grants database was then developed which was updated until 10 February 2010, when the data were locked.

### Assessment of research publications at MakCHS

To determine the range and impact of research publications, a list of MakCHS faculty was generated and an online author search was performed using the following databases: PubMed, Medline, African Indicus Medicus, African Journals Online, Biomed CINAHL, and EMBASE. All articles published between January 1, 2005, and December 31, 2009, were included in these analyses.

A data collection instrument was designed to capture information about the publications, such as names of authors, authorship position, title, journal, date of publication, and HSSP area that the publication addressed. Publications were assigned a primary topic area, based on a list of HSSP priorities that included malaria, HIV/AIDS, TB, maternal and child health, environmental health, and mental health.

These data were entered into formatted Microsoft® Office Excel 2007 spreadsheets and analyzed. Descriptive analyses were performed on both the grants and publication data to generate frequencies, percentages and means. The results were then displayed as tables, bar graphs, and pie charts, or described in text.

## Results

### Research grants

**Figure 1:**
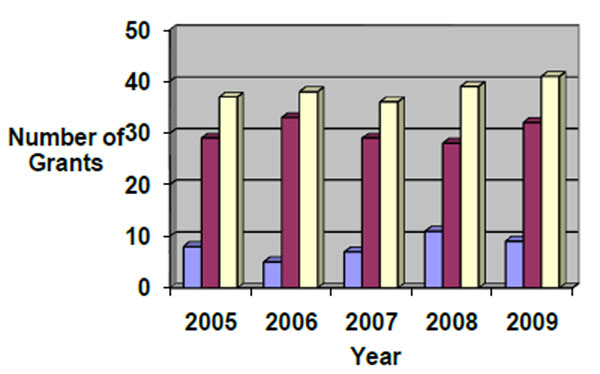
Number of grants at MakCHS by year (2005-2009)

**Figure 2:**
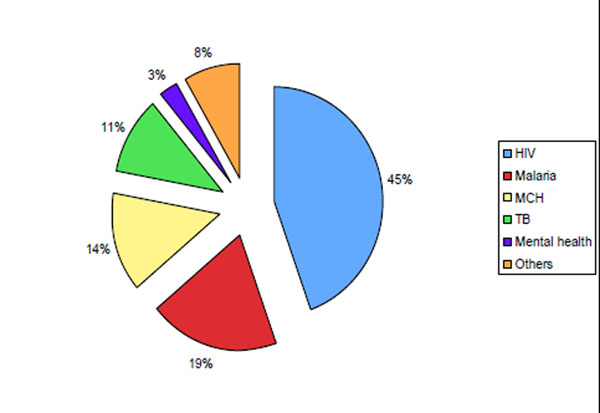
**Major research focus of grants awarded to MakCHS 2005 – 2009. **TB: Tuberculosis; MCH: Maternal and Child Health, Other: socio-cultural, animal and natural products studies.

A total of 58 active grants at the MakCHS were identified between 2005 and 2009, including17 ongoing grants that were awarded before 2005 and 40 new grants awarded during the period of 2005 – 2009, giving an average of 8.0 (SD=2.2) new grants per year. The number of new grants and the overall number of grants per year did not significantly change during the five-year period (Figure 1). The majority of grants funded basic and applied research (36, 62%). Of the remainder, 11 (19%) were training grants and eight (14%) were capacity building grants with research training components. Three (5%) grants were for educational administrative support. Figure 2 shows that the principal focus areas of the 36 research grants awarded to MakCHS were HIV/AIDS (16, 44%), malaria (7, 19%), maternal and child health (5, 14%), tuberculosis (4, 11%) mental health (1, 3%) and other (3, 8%)

**Figure 3:**
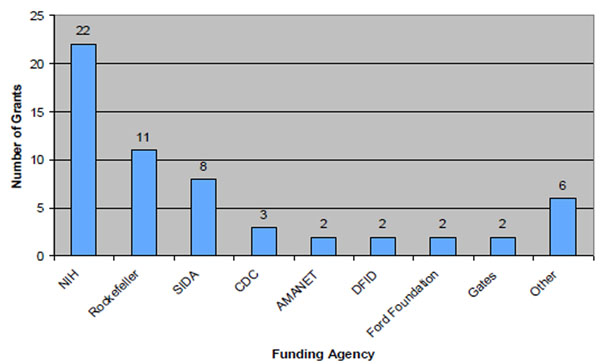
**Major grant sponsors of MakCHS for new and ongoing grants between 2005 -2009**. ‘Other’ Funding agencies included: USAID, Carnegie, EU, and MSI.

North American and European funding agencies were the most common sponsors of grants to MakCHS. Figure 3 shows that the U.S. National Institute of Health provided more than one third of all grants (38%), typically for basic and applied research. Center for Disease Control and Prevention, on the other hand, provided over half of all grant money identified. Other grant sponsors included the African Malaria Network Trust, the Bill and Melinda Gates Foundation, Canadian International Development Agency, Carnegie Corporation, Department for International Development, Doris Duke Charitable Foundation, European and Developing Countries Clinical Trials Partnership, the Ford Foundation, the Millennium Science Initiative , Netherlands Directorate-General for Development Cooperation, Norwegian Agency for Development, Pfizer, the Rockefeller Foundation**,** Swedish International Development Cooperation Agency, and the U.S. Agency for International Development (USAID). MakCHS academic staffs were identified as Principal Investigators (PIs) for only 22 (38%) of the grants. Twenty-four grants (41%) had foreign PIs and data were not available to identify the PI for 12 (23%) of the grants. Budget information was available for only 18 (31%) of the 58 grants. Based on the available data, this study captured a total of US$126.2 million in grant support, of which US$37.5 million were new grants awarded between 2005 and 2009.

### Research publications

**Figure 4:**
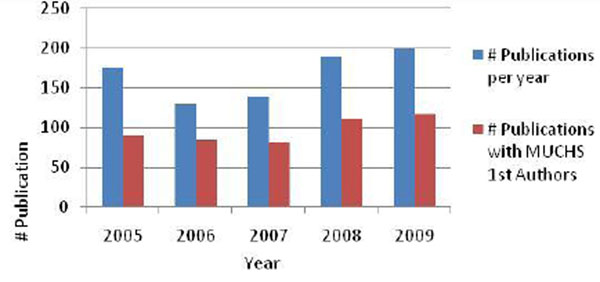
Research publications per year and MakCHS first authors per year from 2005-2009

A total of 837 publications authored by MakCHS faculty were identified during the study period, with an average of 167 publications per year. Figure 4 demonstrates that the number of publications per year did not significantly change during the study period. Almost all of the publications (817, 97.6%) were original research journal articles. These publications appeared in journals with a median impact factor of 2.72 (range of 0.96 to 28.4, impact factors being available from all but two of the journals). In addition to original research, the publications included 17 editorial reviews, two letters to the editors, and one comment. As illustrated in Table 1, the majority of publications (66%) addressed priorities of the Ugandan HSSP. One third or 295 of the papers (35.2%) related to HIV/AIDS, 114 (13.6%) to malaria, 85 (10.2%) to maternal and child health, 43 to tuberculosis, and 24 (2.9%) to mental health issues. More than half (485, 57.9%) of the publications were first-authored by MakCHS faculty (see Figure 4).

**Table 1:** Focus Area of 837 publications from MakCHS from 2005-2009

Focus area of the publications	Number	Percentage (%)
HIV/AIDS	295	35.2
Malaria	114	13.6
Maternal Child Health	85	10.2
Basic cellular research/genetics	49	5.9
Tuberculosis	43	5.1
Socio-cultural Issues	39	4.7
Other non-communicable diseases	36	4.3
Other infectious diseases	25	3.0
Mental Health	24	2.9
Environmental health	21	2.5
Cancer	20	2.4
Medical education	16	1.9
Cardiovascular disease	13	1.6
Other (Animal studies, herbal medicinal products, editorials and letters)	57	6.7

Of the 837 articles the top 10 most productive first authors included five from Makerere and five from outside Uganda. All of the authors outside Makerere wrote articles related to HIV/AIDS research based on the grants implemented at Makerere. Of the five authors from MakCHS two described research in malaria, one in community interventions, one in primary care and mental health, and one in cancers associated with HIV/AIDS. These Makerere authors better reflected the diversity of the HSSP priorities. Of the top 10 most productive last authors only 2 were from Makerere, and, again reflecting the grants, the majority described research in the areas of HIV/AIDS and malaria. Based on the country location of the corresponding author it is clear that MakCHS has many foreign partners. About half (431 articles) of the corresponding authors are from outside Uganda: four countries in the Americas, 12 countries in Europe, three in Asia, Australia, and 8 in Africa. The largest number (157 articles) coming from the US.

## Discussion

Our analysis of MakCHS’ research grant and publication portfolio highlighted a number of key findings which can inform future strategies for optimizing the College’s research capacity. Our methods and finding may also be helpful to other African research institutions. In general, our study found that MakCHS has a diverse scope of health research activities that are productive and contributing significantly to the peer reviewed health literature that informs health policy and future research. In addition to a large number of research grants, MakCHS was particularly productive in the number of peer-reviewed research manuscripts published.

The study also found that grants and publications at MakCHS over the past five years were generally well-aligned with Uganda’s HSSP priorities. However, the major focus of MakCHS research activities, reflected by the number of grants, amount of money and publications, is HIV/AIDS. There are twice as many grants for HIV as for malaria. However, malaria is the most commonly reported disease in Uganda; clinically diagnosed malaria is the leading cause of morbidity and mortality [1-3]. Malaria accounts for 25 to 40% of outpatient visits, 15 to 20% of hospital admissions, and 9 to 14% of hospital deaths. Almost half of all deaths among child under 5 years is attributed to clinical malaria [3]. In 2009 there were 64,000 death from HIV and 116,800 deaths from malaria of which 100,000 were children under 5 [2,3]. Our analyses demonstrated that the priorities and opportunities for research may not reflect completely the relative impact of health issues. While HIV/AIDS may only be at best second to malaria in terms of health priority in Uganda, MakCHS has twice as many research grants for HIV/AIDS research as for malaria research.

MakCHS has collaborations with several institutions both internationally and locally. A number of key international MakCHS collaborations identified in this study have specific research areas of interest, such as the Case Western Reserve University collaboration focused on TB, the Johns Hopkins University focused on HIV/AIDS, and the University of California at San Francisco on malaria [4,5]. As such this may influence the funding and publication from MakCHS faculty.

In addition, it is may be that the focus of research at MakCHS, to some degree, may be donor-driven and reflect the expanded opportunities for research grants in areas such as HIV/AIDS. The major sponsors of grants at MakCHS were found to be the North American and European funding agencies, with United States funding agencies leading in both number of grants and total funding. In the past five years, these and other foreign donors have directed large amounts of money to MakCHS, while at the same point in time, public funding for medical and health-related research in Uganda has remained very low at 0.4% of GDP for all government research funding with only a portion of this for medicine and health [6]. At the same time, foreign sponsors and collaborators are not supporting local PI leadership, probably because the grants are initiated in the funding countries. The current arrangement harbours increasing risks for the research priorities and activities at MakCHS to become skewed towards external, foreign interests. The kinds of research most valuable to external agents may not be those most valuable to the Ugandan public, as stipulated in the HSSP. The conflict of interest may arise from the fact that funding agencies, through the exercise of strategic funding, are able to exert controlling influence in various fields of endeavour, and international health provides no exception to this general rule. For example, currently, international health professionals are confronted with two contending conceptualizations of health: 1) a broad health oriented concept reflected primarily as a social product and hence leads to social-oriented solutions, and 2) a disease oriented concept which leads to a biotechnical, individual-oriented disease treatment solution. If MakCHS is to be responsive to the HSSP selection of health priorities then the wider definition of health needs be selected, and this frequently does not match with external funders’ interests. This choice represents a particularly difficult dilemma to international health professionals who are dependent on external funding agencies for their support [7]. However, despite the fact that the major sources of grants at MakCHS are external agents, it is reassuring to find that most grants fund activities are in line with the Uganda’s HSSP. This may be attributed, in part, to the call by the Global Forum to address the 10/90 gap [8] to shift health research priorities from problems of industrialized countries to those affecting populations in developing countries, which has led to redirection of international research priories to try to correct this gap, and to build capacity in the countries of greatest need [9].

Our analysis identified more than US$ 126.2 million in grant support was provided to MakCHS between 2005 and 2009. However, because of the difficulties in obtaining complete data on all grants, this is clearly an underestimation. We were only able to access amount of grant support on 18 of the 58 grants. Our analysis demonstrated that key information, including even the name of the PI were often not available for a large number of grants provided to MakCHS. It was easiest to access funding information on grants that were channelled to MakCHS directly. We observe here that as a result of the fragmented management of research funds at Makerere [4] retrieval of information on grants was problematic.

Although the number of grants and publications at MakCHS may look high it is not possible to draw any conclusions on these as there is no standard of comparison. However, in both the grants and publications, the role of the MakCHS faculty in the leadership and management of these grants and publications can be improved. Our analyses demonstrated that the majority of grant PIs were not MakCHS faculty members, but the majority of publications were first-authored by MakCHS. The findings that key budget information was lacking and that the majority of PIs were foreign is consistent with a prior report on research collaborations between Makerere University and Swedish Universities where it was observed that grant management was weak due to a lack of a central grants management system [5]. Our findings support their recommendation for establishment of an efficient, reliable, centralized MakCHS grants management administrative structure. To increase the number of grants lead by MakCHS faculty, a grants management office would also need to provide mentorship and support of junior academic faculty.

## Conclusions

MakCHS research capacity is broad and has been highly productive over the past five years. The research grants and publications at MakCHS are generally well-aligned with the Ugandan Health Ministry priorities. Greater efforts to establish centralized and efficient grants management procedures are needed. In addition, greater efforts are needed to expand the MakCHS faculty’s capacity for leadership of grants, as well as their contribution to lead research publications.

## List of abbreviations used

MakCHS: Makerere University College of Health Sciences; HSSP: Health Sector Strategic Plan; USAID: U.S. Agency for International Development; PI: Principal Investigator

## Competing interests

The authors declare that they have no competing interests.

## Authors' contributions

ZN was involved in the conceptualization of the assessment, analysis of the data and writing of the manuscript. PK, DK and JK were involved in the conceptualization of the assessment and data analysis. SG was involved in the data analysis and drafting the manuscript. RB and CO were involved in the conceptualization of the assessment, data analysis and helped in drafting and final approval of the manuscript. All authors read and approved the final manuscript.
